# Design of a Novel Nanosensors Based on Green Synthesized CoFe_2_O_4_/Ca-Alginate Nanocomposite-Coated QCM for Rapid Detection of Pb(II) Ions

**DOI:** 10.3390/nano12203620

**Published:** 2022-10-15

**Authors:** Wafa Al-Gethami, Dalal Alhashmialameer, Noha Al-Qasmi, Sameh H. Ismail, Ahmed H. Sadek

**Affiliations:** 1 Chemistry Department, Faculty of Science, Taif University, Al-Hawiah, Taif City P.O. Box 11099, Saudi Arabia; 2 Faculty of Nanotechnology for Postgraduate Studies, Sheikh Zayed Campus, Cairo University, 6th October City, Giza 12588, Egypt; 3 Zewail City of Science, Technology and Innovation, 6th October City, Giza 12578, Egypt

**Keywords:** QCM, sensor, Pb(II), CoFe_2_O_4_ nanoparticles, CoFe_2_O_4_/Ca-Alg nanocomposite, green synthesis

## Abstract

Pb(II) is a significant contaminant that is known to have negative effects on both humans and animals. Recent industrial operations have exacerbated these consequences, and their release of several contaminants, including lead ions, has drawn attention to the potential effects on human health. Therefore, there is a lot of interest in the rapid, accurate, and selective detection of lead ions in various environmental samples. Sensors-based nanomaterials are a significant class among the many tools and methods developed and applied for such purposes. Therefore, a novel green synthesized cobalt ferrite (CoFe_2_O_4_) nanoparticles and functionalized CoFe_2_O_4_/Ca-alginate nanocomposite was designed and successfully synthesized for the fabrication of nanoparticles and nanocomposite-coated quartz crystal microbalance (QCM) nanosensors to detect the low concentrations of Pb(II) ions in the aqueous solutions at different temperatures. The structural and morphological properties of synthesized nanoparticles and nanocomposite were characterized using different tools such as X-ray diffraction (XRD), N_2_ adsorption–desorption isotherm, dynamic light scattering (DLS), zeta potential analyzer (ζ-potential), atomic force microscopy (AFM), scanning electron microscopy (SEM), transmission electron microscopy (TEM), and energy-dispersive X-ray spectroscopy (EDX). The QCM results revealed that the green synthesized CoFe_2_O_4_ nanoparticles and functionalized CoFe_2_O_4_/Ca-alginate nanocomposite-coated QCM nanosensors exhibited high sensitivity, stability, and rapid detection of Pb(II) ions in the aqueous solutions at different temperature. The lowest detection limit for Pb(II) ions in the aqueous solutions could reach 125 ng, which resulted in a frequency shift of 27.49 ± 0.81, 23.63 ± 0.90, and 19.57 ± 0.86 Hz (Δ*f*) for the QCM detector coated with green synthesized CoFe_2_O_4_ nanoparticles thin films, and 25.85 ± 0.85, 33.87 ± 0.73, and 6.87 ± 0.08 Hz (Δ*f*) for the QCM detector coated with CoFe_2_O_4_/Ca-Alg nanocomposite thin films in a real-time of about 11, 13, and 13 min at 25 °C, 35 °C, and 45 °C, respectively. In addition, the resonance frequency change results showed the superiority of functionalized CoFe_2_O_4_/Ca-alginate nanocomposite coated QCM nanosensor over CoFe_2_O_4_ nanoparticles towards Pb(II) ions detecting, which attributed to the beneficial properties of alginate biopolymer.

## 1. Introduction

Lead is a chemical element that occurs naturally. Lead is a typical industrial metal that is now present in large quantities in the air, water, soil, and food supply. Lead is mostly used in storage batteries (72%), gasoline additives and other chemicals (13%), ammunition (shot and bullets, 4%), solder (2%), and other purposes (9%). More than 3 million tons are produced worldwide each year [1,2,3,4]. Lead, in its aqueous soluble form, is one of many metal ions that is regarded as a growing water contaminant. Beyond the permitted limit, these heavy metals can pose substantial risks to both human and animal health. Higher concentrations of lead in drinking water have been linked to conditions such as hepatitis, encephalopathy, anemia, and nephritic syndrome. The neurological, kidney, bone, and blood circulation systems are primarily impacted by lead toxicity. The maximum allowable lead concentration in drinking water was advised to be 0.01 mg/L by the World Health Organization (WHO) standards for drinking water quality standard [5,6,7,8,9].

The conventional techniques that have been used to remove metal ions include chemical precipitation, ion exchange, membrane filtration, reverse osmosis, ion exchange, electrodialysis, and adsorption. However, several of these conventional approaches have drawbacks, such as high prices and lower efficacy at low metal concentrations of 1–100 mg/L [10,11,12]. Therefore, the development of straightforward, quick, and affordable heavy metal detection techniques is a significant challenge for scientists since heavy metal ions have a detrimental effect on both human health and the environment, even at low concentrations [13,14].

The detection of heavy metal ions in aqueous solutions is a target of particular importance in environmental analysis, early prevention, and control of pollution. Thus, a difficult challenge for environmental pollutant detection is the development of extremely sensitive and selective chemo-sensors or biosensors for heavy metal ions in the aqueous solutions. Among these sensors, the quartz crystal microbalance (QCM) sensors-based nanomaterials showed a sensitive enhanced chemical change for many heavy metal ions and thus might be considered a useful testing instrument [15,16,17]. The sensor based on QCM is a significant and promising sensing method for the online environmental analysis and real-time detection of traced heavy metal ions in aqueous solutions. A QCM resonator can sensitively and precisely monitor the change in quartz resonance frequency caused by the mass adsorbed on the piezoelectric quartz crystal. The QCM-based sensor has been thoroughly investigated for detecting the trace mass changes in the nano-gram range, which are absorbed onto the electrode surface of the quartz crystal in the air or a liquid. The sensor is very sensitive and is based on quartz crystals. For the very sensitive and precise detection of heavy metal ions in aqueous environments, several QCM sensors have been designed and coated with layers of tiny molecules [14,15,16,17,18]. However, the drawbacks of using tiny molecules in fabrication devices make it difficult to design QCM sensors for use in continuous media. The functional polymeric materials offer an alternate method for modifying the surface characteristics of quartz crystals and expanding the scope of QCM sensors’ applications [19,20,21,22].

Due to their distinctive architectures and superior optical, electrical, and catalytic characteristics, nanomaterials have received extensive research in optical, electronic, and electrochemical sensors for the detection of water contaminants. The nanomaterial-based sensors have considerable potential for detecting water contaminants and surpass traditional sensors and technologies in different aspects, such as high sensitivity, quick response, and ease of use [23,24,25]. Regarding their superior characteristics to those displayed by the traditional materials with grains size > 10 µm, magnetic spinel ferrite nanoparticles have garnered enormous attention over the past two decades. They have a significant potential for use in a variety of applications, including magnetic recording, magnetic energy storage, catalysis, biomedicine, and wastewater treatment, because of their special composition and microstructure. The inverse spinel cobalt ferrite (CoFe_2_O_4_) is one of the most intriguing members of the spinel ferrites family. It has excellent physical and chemical stability, large anisotropy, saturation magnetization, and tunable coercivity, making it a convenient candidate for environmental applications [26,27,28].

It is widely known that the synthesis process has a significant impact on the composition, structure, and morphology of the magnetic ferrite nanoparticles, as well as indirectly, on their properties [29]. Biological synthesis is a straightforward, affordable, and environmentally friendly process that uses bacteria, algae, and plant extracts. It has advantages over other methods since it does not require dangerous chemicals, high temperatures, or high pressures [30]. The plant extracts have promising advantages due to their low toxicity, accessibility, safety, ease of improvement, extravagant procedure of retaining cell structures, and plenty of active agents that can enhance the reduction of metal ions. The plant extract also contains a variety of functional primary chemicals, such as polyphenols, amino acids, proteins, terpenoids, ketones, and aldehydes, that impact the reduction and capping of the nanoparticles to produce the required shape and size [31,32,33]. Clove (*Syzygium aromaticum*) is the aromatic flower buds of an Indonesian plant and was considered an ideal choice for biological activities due to it being a major source of phenolic compounds [34].

On the other hand, cobalt ferrite gains better characteristics when mixed with biopolymers like sodium alginate. The biopolymer alginate (Alg) is extracted from brown seaweeds, which is a non-toxic, biocompatible, and biodegradable polymer. It consists of blocks with 1–4 linked connected α-L-guluronic and β-D-mannuronic acids. Alg is capable of easily forming cross-linked gel matrices in the presence of divalent cations, particularly the Ca^2+^ ion. As a result, these Ca-cross-linked Alg matrices can be employed to generate gel phase adsorbents, which are simpler to handle than powder materials. The adsorbing efficiency of Alg-based formulations is particularly related to the presence of carboxylic groups in the Alg structure that enable it to form complexes with metal ions in aqueous solutions [35,36].

Moreover, the selectivity of nanoparticles- and nanocomposites-based sensors could increase by modifying or developing a new sensor to determine the target analyte in real samples [37,38]. This would be more applicable to the magnetic CoFe_2_O_4_/Ca-Alg nanocomposite to a great extent due to the considerable advantages of alginate biopolymer owing tremendous benefits of alginate biopolymer, such as its availability, biocompatibility, biodegradability, low cost, high porosity and permeability, non-toxicity, and large specific surface area [39,40]. Furthermore, these polymers’ hydrophilicity allows for higher water fluxes than many synthetic polymers. Therefore, they are commonly used for water purification purposes. In addition, the hydrated molecular structure of alginate hydrogel allows for absorbing a large amount of water and swelling to larger volumes. These properties nominate the CoFe_2_O_4_/Ca-Alg nanocomposite to be an efficient nanosensor material [41,42].

Therefore, the main objective of this work was to demonstrate the first green synthesis of pristine cobalt ferrite (CoFe_2_O_4_) nanoparticles using clove (*Syzygium aromaticum*) extract and then to functionalize the as-prepared CoFe_2_O_4_ nanoparticles with Ca-alginate biopolymer to produce a CoFe_2_O_4_/Ca-Alg nanocomposite. The developed nanomaterial and its nanocomposite were subjected to different characterization tools such as XRD, BET, DLS, zeta potential, AFM, SEM, TEM, and EDX to reveal their structure and morphology for size and shape. Subsequently, the CoFe_2_O_4_ nanoparticles and CoFe_2_O_4_/Ca-Alg nanocomposite were investigated as novel nanomaterial-based-QCM sensors for the rapid and efficient detection of Pb(II) ions in the aqueous solutions at different temperatures.

## 2. Materials and Methods

### 2.1. Materials

Iron(III) chloride hexahydrate (FeCl_3_·6H_2_O, ACS reagent, 97%), cobalt(II) chloride hexahydrate (CoCl_2_·6H_2_O, ACS reagent, 98%), sodium hydroxide (NaOH reagent grade, ≥98%, pellets (anhydrous)), sodium alginate (C_6_H_9_NaO_7_, molecular weight of 216.12 g/mol, viscosity 5.0–40.0 cps (c = 1%, H_2_O @ 25 °C)), calcium chloride (CaCl_2_, anhydrous, granular, ≤7.0 mm, ≥93.0%), and lead(II) nitrate (Pb(NO_3_)_2_, ACS reagent, ≥99.0%) were purchased from Sigma-Aldrich (Munich, Germany). All chemicals were used as received without any more purification. Double-distilled water was used for all solutions preparation and rinsing throughout this work.

### 2.2. Preparation of the Clove Leaves Extract

After being bought from a local market, the clove (*Syzygium aromaticum*) was initially washed with tap water. After that, they were rinsed with double distilled to remove waste and impurities. They were then allowed to dry naturally for five days. The leaves extract was made by mixing 6 g of dried leaves with 100 mL of double-distilled water. It was then heated at 60 °C for 30 min. Afterward, the clove leaf extract was filtered and stored for future investigation [43].

### 2.3. Green Synthesis of CoFe_2_O_4_ Nanoparticles

With a few minor modifications, the co-precipitation method was used to synthesize spinel CoFe_2_O_4_ nanoparticles. Double-distilled water was used to dissolve 5 g of FeCl_3_·6H_2_O and 3 g of CoCl_2_·6H_2_O. The mixture was then heated on a hot plate for about 15 min at 50 °C. Then, 10 mL of the clove leaves extract was added to the chloride solution while stirring vigorously. By adding drops of a 0.5 M NaOH solution to the mixture, the pH was raised to 10. The mixture was then stirred for 2 h at 60 °C. The resulting nanoparticles were calcined for 2 h at 600 °C after being washed with double-distilled water.

### 2.4. Preparation of CoFe_2_O_4_/Ca-Alg Nanocomposite

CoFe_2_O_4_/Ca-Alg nanocomposite was prepared via the ionotropic gelation mechanism. An amount of 0.025 g of CoFe_2_O_4_ was sonicated in 50 mL of distilled water for 20 min. Subsequently, 1 g of sodium alginate was added to the CoFe_2_O_4_ nanoparticles solution and stirred for 1 h followed by sonicating for 20 min. Afterward, calcium chloride solution (2M) was added to the mixture and stirred for 1 h. The mixture was left in the refrigerator for 2 days. The formed nanocomposite was filtrated and washed 3 times with distilled water. The prepared CoFe_2_O_4_/Ca-Alg nanocomposite was then dried in an oven at 60 °C for 2 days.

### 2.5. Characterization

The composition and phase identification of both green synthesized CoFe_2_O_4_ nanoparticles and CoFe_2_O_4_/Ca-Alg nanocomposite was obtained using X-ray diffraction (EQUINOX 1000, Thermo Scientific CO., Lafayette, CO, USA). The employed X-ray source was Cu Kα radiation with a current of 31 mA and an applied voltage of 33 kV. The 2θ angles ranged from 5° to 80° with a scan speed of 0.1°/min. N_2_ adsorption–desorption analyzer (Nova Touch 4L, Quanta Chrome, Boynton Beach, FL, USA) was used to determine the surface area and pore size of the green synthesized CoFe_2_O_4_ nanoparticles and CoFe_2_O_4_/Ca-Alg nanocomposite according to the BET and DA methods, respectively. Prior to performing the BET test, the green synthesized CoFe_2_O_4_ nanoparticles and CoFe_2_O_4_/Ca-Alg nanocomposite was degassed at 75 °C for 2 h to dispose of any moisture or gas molecules on the surface of the tested materials. In addition, the zeta seizer instrument (NanoSight NS500, Malvern Panalytical, Malvern, UK) was used to determine the particle size (DLS method) and surface charge of the green synthesized CoFe_2_O_4_ nanoparticles and CoFe_2_O_4_/Ca-Alg nanocomposite. Moreover, in order to identify the shape and morphology of green synthesized CoFe_2_O_4_ nanoparticles and CoFe_2_O_4_/Ca-Alg nanocomposite, the topographic properties of the prepared CoFe_2_O_4_ nanoparticles and CoFe_2_O_4_/Ca-Alg nanocomposite were investigated using AFM, SEM, and TEM instruments. The AFM equipment (5600LS, Agilent technology firm, Santa Clara, CA, USA) was used to provide 2D and 3D topographic images for the synthesized materials. Before the AFM analyses, the samples were first introduced to ultrasonic waves for two hours using an ultrasonic probe sonicator (UP400S, Hielscher, Oderstraße, Teltow, Germany) for 20 min at 59 kHz, 83% amplitude, and 0.79 cycles. Finally, a thin film of the samples was created under a vacuum using a spin coater instrument (WS-650Sz, Laurell, North Wales, PA, USA) at 600 rpm. Additionally, Gwyddion software (supported by the department of nanometrology and technical length, Czech Metrology Institute, Okružní, Czech Republic) was utilized to evaluate the AFM outcomes. The AFM images and data profiles were obtained at 100 nm × 67 nm using tapping mode imaging (Al tap, 0.4 In/S speed, I. gain 0.4, and P. gain 20). An SEM instrument (JEOL, JSM-6701F Plus, Peabody, MA, USA) equipped with energy dispersive X-ray spectrometry (EDX) for elemental analysis and TEM (JEOL, JEM-2100 high-resolution, Peabody, MA, USA) were devoted to giving information on the size, shape, and surface morphology of the green synthesized CoFe_2_O_4_ nanoparticles and CoFe_2_O_4_/Ca-Alg nanocomposite. The SEM images were taken at an acceleration voltage of 10 kV and magnification of 3000 Kx. Before TEM investigation, the CoFe_2_O_4_ nanoparticles and CoFe_2_O_4_/Ca-Alg nanocomposite were mixed with double-distilled water and were sonicated for 20 min using an ultrasonic probe sonicator at a frequency of 55 kHz, an amplitude of 55%, and a cycle of 0.55. Then drops with 5 to 10 microns of the dispersed mixture were dropped over a carbon-coated copper grid, which was subsequently submitted to the TEM test.

### 2.6. Quartz Crystal Microbalance (QCM)

The sensitivity, selectivity, and stability of the produced CoFe_2_O_4_ nanoparticles and CoFe_2_O_4_/Ca-Alg nanocomposite-coated QCM nanosensors for the detection of Pb(II) ions in an aqueous solution were assessed using a quartz crystal microbalance with dissipation monitoring (QCM, Q-senses, Biolin Scientific, Linthicum Heights, MD, USA). Gold-coated AT-cut quartz crystals (Q-Sense) were utilized with a fundamental frequency (*f*_0_) of 5 MHz. Before measurement and spin coating, the quartz crystals were rinsed with double-distilled water and dried.

In the experiment, the flow cell of the QCM-D was fitted with a recently cleaned quartz crystal. The quartz crystal’s frequency shift was measured in order to compare them to the manufacturer’s calibration standards. The QCM-D flow cell was flushed with double-distilled water until a steady baseline was attained. Afterward, 50 µg/L of green synthesized CoFe_2_O_4_ nanoparticles or CoFe_2_O_4_/Ca-Alg nanocomposite was dispersed in 20 mL of doubled distilled water flow on the QCM chip surface at a speed of 0.25 mL per min until stable baseline frequency was obtained, which indicate the successful coating of CoFe_2_O_4_ nanoparticles or CoFe_2_O_4_/Ca-Alg nanocomposite on the QCM chip surface. Subsequently, an aqueous solution of Pb(II) ions with a given concentration was flushed on the surface of fabricated CoFe_2_O_4_ nanoparticles or CoFe_2_O_4_/Ca-Alg nanocomposite-coated QCM nanosensors.

Pb(II) ion adsorption causes a shift in frequency that is measured against time. Adsorption vs. time measurements typically lasted a few minutes until equilibrium in adsorption was evident. A peristaltic pump (ISM 930, IPC, Ismatec, Wertheim-Mondfeld, Germany) was used to control the flow rate in each experiment, which was maintained at 0.25 mL/min. All experiments were conducted in a cell at different temperatures of 25 °C, 35 °C, and 45 °C. For the final rinse, double-distilled water was used once more in place of the ion solution. The Pb(II) ion solution concentrations were restricted to a very low value of 125 ng/L (1 µg of Pb(NO_3_)_2_ dissolved in 200 mL double-distilled water); at this concentration, the viscosities and densities of the metal ion solutions remain constant.

## 3. Results and Discussion

### 3.1. Characterization of Green Synthesized CoFe_2_O_4_ Nanoparticles and CoFe_2_O_4_/Ca-Alg Nanocomposite

#### 3.1.1. XRD

**Figure 1:**
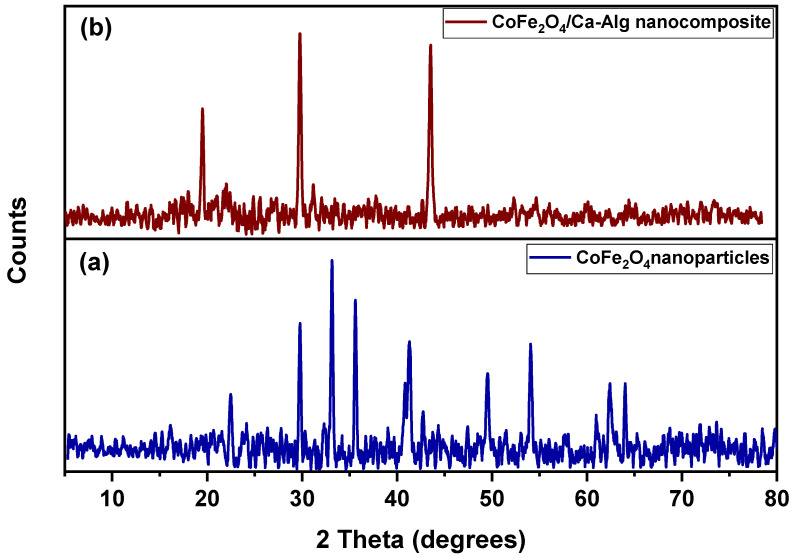
Shows the XRD patterns of (**a**) green synthesized CoFe_2_O_4_ nanoparticles and (**b**) CoFe_2_O_4_/Ca-Alg nanocomposite.

Figure 1a,b show the XRD patterns of green synthesized CoFe_2_O_4_ nanoparticles and the CoFe_2_O_4_/Ca-Alg nanocomposite, respectively. For CoFe_2_O_4_ nanoparticles, it could observe the reflection planes of (220), (311), (422), (511), (440), and (533) plans that correspond to the 2θ of 30.25°, 35.63, 53.75°, 57.28°, 62.91°, and 74.44°, respectively. These results confirm the successful green synthesis of CoFe_2_O_4_ nanoparticles, which is present in a face-centered cubic lattice structure. The high crystallinity of green synthesized CoFe_2_O_4_ was delivered in sharp and narrow diffraction peaks with good intensities [44,45]. In addition, some impurities peaks were displayed in the diffractogram, which may be attributed to the clove leaf extract compounds that coated the prepared CoFe_2_O_4_ nanoparticles during the synthesis process. For CoFe_2_O_4_/Ca-Alg nanocomposite, it could be observed the presence of a few peaks differ from that of the CoFe_2_O_4_ nanoparticles pattern and the disappearance of the featured peaks of CoFe_2_O_4_ nanoparticles. These results demonstrate the successful coating of CoFe_2_O_4_ nanoparticles by Ca-Alg biopolymer, and the observed peaks may be attributed to the small contributions that originated from the substrates.

#### 3.1.2. BET Surface Area and Porosity Properties

**Figure 2:**
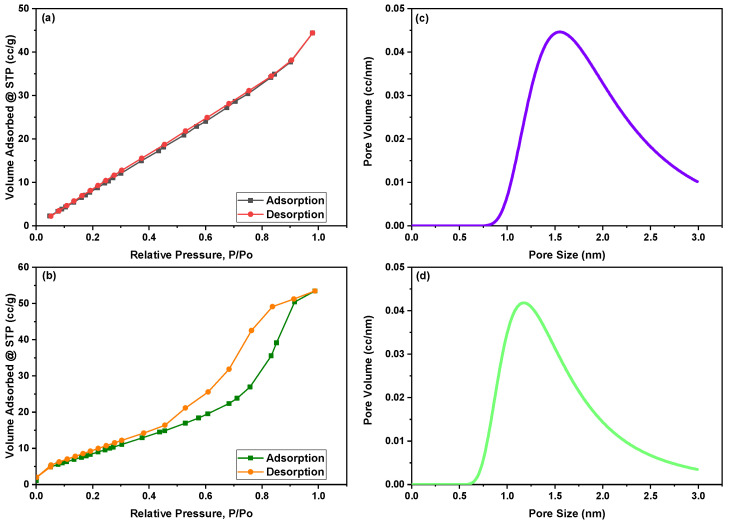
Illustrates (**a**,**b**) the N_2_ adsorption–desorption isotherm curves of green synthesized CoFe_2_O_4_ nanoparticles and CoFe_2_O_4_/Ca-Alg nanocomposite according to the BET method, respectively, and (**c**,**d**) pore size/volume as determined by the DA method for green synthesized CoFe_2_O_4_ nanoparticles and CoFe_2_O_4_/Ca-Alg nanocomposite, respectively.

Figure 2a,b displays N_2_ adsorption–desorption isotherm curves of green synthesized CoFe_2_O_4_ nanoparticles and CoFe_2_O_4_/Ca-Alg nanocomposite as measured by the BET method, respectively. While Figure 2c,d shows the pore size distribution of both green synthesized CoFe_2_O_4_ nanoparticles and CoFe_2_O_4_/Ca-Alg nanocomposite, respectively. According to the IUPAC classification of adsorption isotherms, the isotherm curve of CoFe_2_O_4_ nanoparticles showed a typical type three (III) class, which does not have the “sharp knee” shape indicating that stronger adsorbate–adsorbate interactions than adsorbate–adsorbent interactions [46]. The green synthesized CoFe_2_O_4_ nanoparticles displayed an H3 hysteresis type, in which the pores have a wedge-shaped pore, according to de Boer’s classification of hysteresis loops [47]. The isotherm curve of CoFe_2_O_4_/Ca-Alg nanocomposite has exhibited type four (IV) of adsorption isotherm, which reveals non-porous or microporous adsorbents with unlimited monolayer–multilayer adsorption. In this isotherm, the adsorption volume quickly increases at low relative pressures due to contact of the adsorbate molecules with the higher energetic section followed by the interaction with the less energetic section. Following the completion of the monolayer formation of the adsorbed molecules, multilayer formation begins to occur in accordance with the “sharp knee” of the isotherm. In contrast, a sudden rise signal indicates the bulk condensation of adsorbate gas to liquid as the relative pressure approaches unity. Moreover, the synthesized CoFe_2_O_4_/Ca-Alg nanocomposite displayed an H2 hysteresis type, in which the pores have an inkbottle-shaped pore and are associated with capillary condensation phenomena in mesoporous structures. This hysteresis loops type indicates the presence of complex pore networks. A summary of area, volume, and pore size results is tabulated in Table 1.

#### 3.1.3. DLS and Zeta Potential

DLS measurements were used to determine the particle size of green synthesized CoFe_2_O_4_ nanoparticles and CoFe_2_O_4_/Ca-Alg nanocomposite. The average size measurements were found to be 75 and 90 nm for green synthesized CoFe_2_O_4_ nanoparticles and CoFe_2_O_4_/Ca-Alg nanocomposite. All the suspensions were comparatively monodispersed and induced good colloidal stability; the analysis found a unimodal size distribution with polydispersity indices. The observed increase in the average size of the CoFe_2_O_4_/Ca-Alg nanocomposite demonstrates the effective stabilization of the CoFe_2_O_4_ nanoparticle with alginate biopolymer. Additionally, the average size is a measure of the hydrodynamic size; as such, its value will consider both the existence of nanoparticles and any solvent molecules connected to the tumbling particle.

In order to investigate the stability of green synthesized CoFe_2_O_4_ nanoparticles and CoFe_2_O_4_/Ca-Alg nanocomposite in the aqueous mediums, the ζ-potentials of green synthesized CoFe_2_O_4_ nanoparticles and CoFe_2_O_4_/Ca-Alg nanocomposite were studied at different applied voltage values. The ζ-values were recorded as −12 and −20 mV for green synthesized CoFe_2_O_4_ nanoparticles and CoFe_2_O_4_/Ca-Alg nanocomposite, respectively. It could observe the ζ-value negatively increased for CoFe_2_O_4_/Ca-Alg nanocomposite, which may be attributed to the carboxylic groups contributed by the alginate structure and indicates the good linking of CoFe_2_O_4_ nanoparticles with Ca-Alg binder. The coating of CoFe_2_O_4_ nanoparticles with Ca-Alg biopolymer participated effectively in decreasing aggregation and deposition of NPs.

#### 3.1.4. AFM

**Figure 3:**
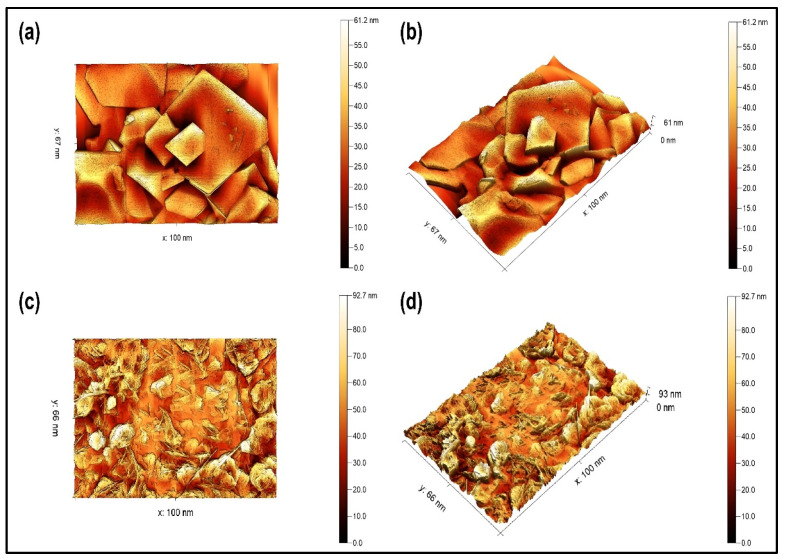
(**a**,**c**) Two-dimensional AFM images of green synthesized CoFe_2_O_4_ nanoparticles and CoFe_2_O_4_/Ca-Alg nanocomposite, respectively; (**b**,**d**) 3D AFM images of green synthesized CoFe_2_O_4_ nanoparticles and CoFe_2_O_4_/Ca-Alg nanocomposite, respectively.

The surface topography of the green synthesized CoFe_2_O_4_ nanoparticles and CoFe_2_O_4_/Ca-Alg nanocomposite was measured using an atomic force microscope. The two- and three-dimensional images of green synthesized CoFe_2_O_4_ nanoparticles and CoFe_2_O_4_/Ca-Alg nanocomposite are shown in Figure 3a–d, respectively. Although some particles slightly varied in size and shape with many overlaps but can be clearly observed the homogeneity and uniformity of CoFe_2_O_4_ nanoparticles. The green synthesized CoFe_2_O_4_ nanoparticles exhibited rhombus bipyramid shapes and formed in good crystallinity with sharp edges. In addition, the nanoparticles fall within the 100 nm scale, with the height of the surface of the particles reaching 61.2 nm. This causes the granular boundary to move more freely, leading to the growth of granule sizes and decreasing the internal and surface defects in the structural texture. This also causes a strong cohesiveness between the granular boundaries. Additionally, the mechanical, electrical, and magnetic properties are improved. Whereas the images of CoFe_2_O_4_/Ca-Alg nanocomposite revealed the surface coating of CoFe_2_O_4_ nanoparticles by Ca-alginate gel, and the alginate network effectively surrounded the majority of CoFe_2_O_4_ nanoparticles with a prominence of some particles on the surface of alginate. Additionally, the coating with alginate further resulted in the appearance of needle shapes and increased the height of the surface of the nanocomposite to 92.7 nm. These results are consistent with the porosity findings from the BET analysis. The average grain size of the samples obtained from AFM images is larger than the particle sizes observed using SEM and TEM measurements, which indicates that each grain is formed by aggregation of a number of nanocrystals.

#### 3.1.5. SEM and TEM

**Figure 4:**
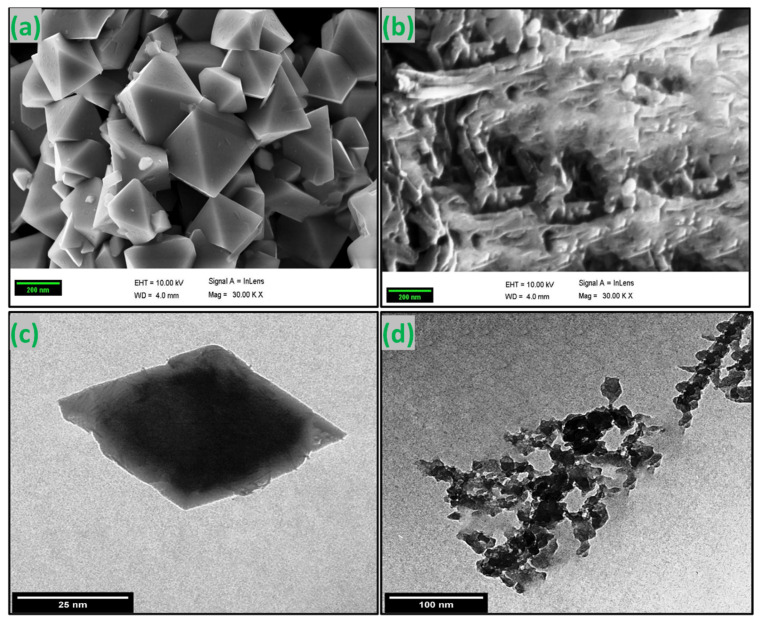
(**a**,**b**) SEM images of green synthesized CoFe_2_O_4_ nanoparticles and CoFe_2_O_4_/Ca-Alg nanocomposite, respectively; (**c**,**d**) TEM images of the green synthesized CoFe_2_O_4_ nanoparticles and CoFe_2_O_4_/Ca-Alg nanocomposite, respectively.

Figure 4a,b show the SEM images of green synthesized CoFe_2_O_4_ nanoparticles and CoFe_2_O_4_/Ca-Alg nanocomposite, respectively. As observed from the image of CoFe_2_O_4_ nanoparticles, the particles were shaped in rhombus bipyramid structures with an excellent degree of crystallinity. In addition, the particles formed separately with sizes in a range of 100 nm and a monodisperse manner. These results additionally support the success of the green synthesis of CoFe_2_O_4_ nanoparticles using the extract of clove. Additionally, the image of CoFe_2_O_4_/Ca-Alg nanocomposite showed a different texture, which is attributed to the gel nature of the alginate-coated CoFe_2_O_4_ nanoparticles. The TEM image of green synthesized CoFe_2_O_4_ nanoparticles further confirmed the dispersity of synthesized particles, where the individual particles formed in regular rhombus bipyramid structures with a size of about 50 nm (Figure 4c). At the same time, the image of the CoFe_2_O_4_/Ca-Alg nanocomposite revealed that the CoFe_2_O_4_ nanoparticles are clearly connected together with the alginate network. Although the size of particles increased because of alginate coating, they are still less than 100 nm (Figure 4d). The results of SEM and TEM are compatible with those obtained by the AFM analysis.

It is obvious that, in addition to particle size and shape, stoichiometry and cation distribution are among the factors that have the greatest impact on the properties of ferrites. Therefore, understanding a material’s chemical composition, structure, and properties is essential to designing it for a particular purpose. In the spinel structure, metal cations fill one of the eight tetrahedral interstices (usually designated as T_d_ or with round brackets) and half of the octahedral interstices (O_h_ or square brackets), which are closely packed together in a cubic arrangement. In a direct or normal spinel (M^II^)[M^III^]_2_O_4_, divalent cations occupy the tetrahedral positions, while in an inverse spinel (M^III^)[M^II^; M^III^]_2_O_4_, trivalent cations replace them. The composition and structure have a direct relationship with the chemical and physical properties. In terms of inversion degree or the proportion of divalent cations in octahedral sites, the bulk cobalt ferrite exhibits an inverse spinel structure, with all Co^II^ cations occupying the octahedral sites while the Fe^III^ cations are equally distributed in the T_d_ and O_h_ sites (γ = 1, γ is the inversion degree or the fraction of divalent cations in octahedral sites). Therefore, the ferrimagnetic behavior of the cobalt ferrite below 860 K is explained by the coupling of the magnetic moments linked to the ions in the Td and Oh sites. In contrast, Co^II^ and Fe^III^ are randomly dispersed when the material is synthesized as a nanostructured material (γ = 0.66). In the literature, the inversion degree of nanostructured CoFe_2_O_4_ prepared using various methods was measured using different techniques (^57^Fe-Mössbauer spectroscopy, EXAFS, neutron diffraction), and the values ranged from 0.68 to 0.76 [48,49,50,51,52].

**Figure 5:**
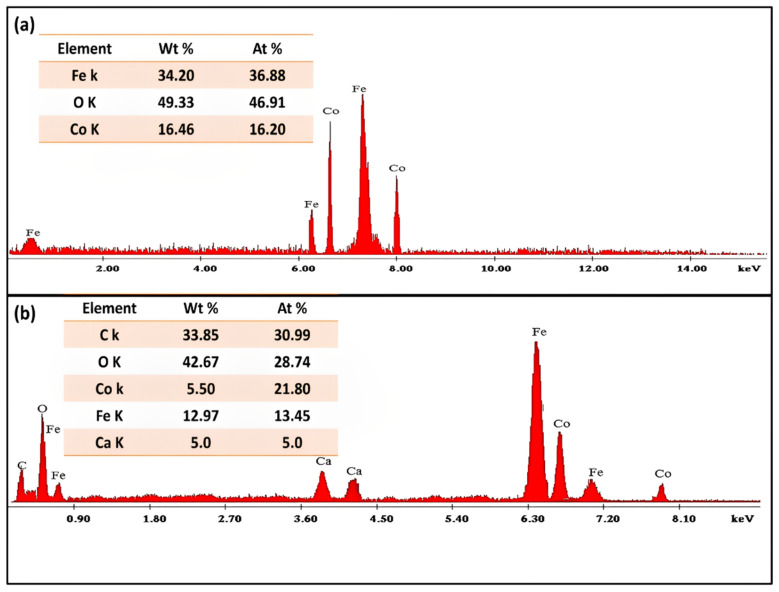
The elemental composition of (**a**) green synthesized CoFe_2_O_4_ nanoparticles and (**b**) CoFe_2_O_4_/Ca-Alg nanocomposite, respectively. Inset: the corresponding table of elements content.

The elemental composition of green synthesized CoFe_2_O_4_ nanoparticles and CoFe_2_O_4_/Ca-Alg nanocomposite was presented in Figure 5a,b as well in the corresponding inset tables. It could be observed that the green synthesized CoFe_2_O_4_ nanoparticles and CoFe_2_O_4_/Ca-Alg nanocomposite samples mainly consist of the three elements (Fe, O, and Co) with wt% of 34.20%, 49.33%, and 16.46% showing an iron/cobalt ratio of 2.1/1 for CoFe_2_O_4_ nanoparticles, and five elements (C, O, Co, Fe, and Ca) with wt% of 33.85%, 42.67%, 5.50%, 12.97%, and 5.0% showing an iron/cobalt ratio of 2.4/1 for CoFe_2_O_4_/Ca-Alg nanocomposite, which represents the major structure of CoFe_2_O_4_ and CoFe_2_O_4_/Ca-Alg, respectively. Where the Fe, O, and Co elements refer to the CoFe_2_O_4_ compound, while the presence of C and Ca elements confirms the successive functionalization of CoFe_2_O_4_ by the alginate biopolymer. These results demonstrated the good formation of both green synthesized CoFe_2_O_4_ nanoparticles and CoFe_2_O_4_/Ca-Alg nanocomposite.

### 3.2. Green Synthesized CoFe_2_O_4_ Nanoparticles and CoFe_2_O_4_/Ca-Alg Nanocomposite-Coated QCM Nanosensors for Detecting Pb(II) Ions in the Aqueous Solutions

Quartz crystal microbalance (QCM)-based heavy metals sensing techniques enable real-time monitoring of the mechanical response of green synthesized CoFe_2_O_4_ nanoparticles and CoFe_2_O_4_/Ca-Alg nanocomposite to Pb(II) ions on the QCM chip surface. However, green synthesized CoFe_2_O_4_ nanoparticles and CoFe_2_O_4_/Ca-Alg nanocomposite precipitate on the QCM chip surface allowed the sense of nano-gram of Pb(II) ions loaded from an aqueous solution in real-time under the controlling of solution temperature using the QCM method. QCM techniques depend on the piezoelectric phenomena of quartz crystal, where the gold electrodes conduct electrical signals to detectors when nano-gram of materials are loaded on the QCM chip surface. The quartz crystals can be eager to the resonance frequency, which is related to the mass (thickness) of the QCM chip. If the mass changes, the resonance frequency (*f*) changes. By real-time monitoring resonance frequency (Δ*f*) changes, detecting small changes in the QCM chip mass (thickness) can be possible. The QCM measurement data enable us to detect nano-scale mass changes such as molecules binding or adsorbing to the surface, which is detected as mass (thickness) increases.

As discussed before, the green synthesized CoFe_2_O_4_ nanoparticles and CoFe_2_O_4_/Ca-Alg nanocomposite possesses a high adsorbent surface area, the availability of more adsorption sites, and a high negative charge density, which can allow easily forming complexes with Pb(II) ions. The green synthesized CoFe_2_O_4_ nanoparticles and CoFe_2_O_4_/Ca-Alg nanocomposite thin films were then fabricated on the quartz crystal with the gold electrode during the flush in of nanoparticles and nanocomposite solutions. The hydrophilic nature of synthesized materials enhances the adhesion of green synthesized CoFe_2_O_4_ nanoparticles and CoFe_2_O_4_/Ca-Alg nanocomposite thin films onto the gold electrode surface especially in the case of CoFe_2_O_4_/Ca-Alg nanocomposite due to the presence of alginate binder. The strong interaction between the green synthesized CoFe_2_O_4_ nanoparticles or CoFe_2_O_4_/Ca-Alg nanocomposite and Au electrode of quartz crystal further assures the stability of formed thin films on the quartz crystal surface in aqueous media, therefore leading to the better coating of the QCM detector. Such green synthesized CoFe_2_O_4_ nanoparticles and CoFe_2_O_4_/Ca-Alg nanocomposite-coated nanosensors were applied to detect the Pb(II) ions in the aqueous solutions by using QCM. The QCM results indicate that the green synthesized CoFe_2_O_4_ nanoparticles and CoFe_2_O_4_/Ca-Alg nanocomposite-coated QCM nanosensors can easily and rapidly adsorb or form complexes with Pb(II).

**Figure 6:**
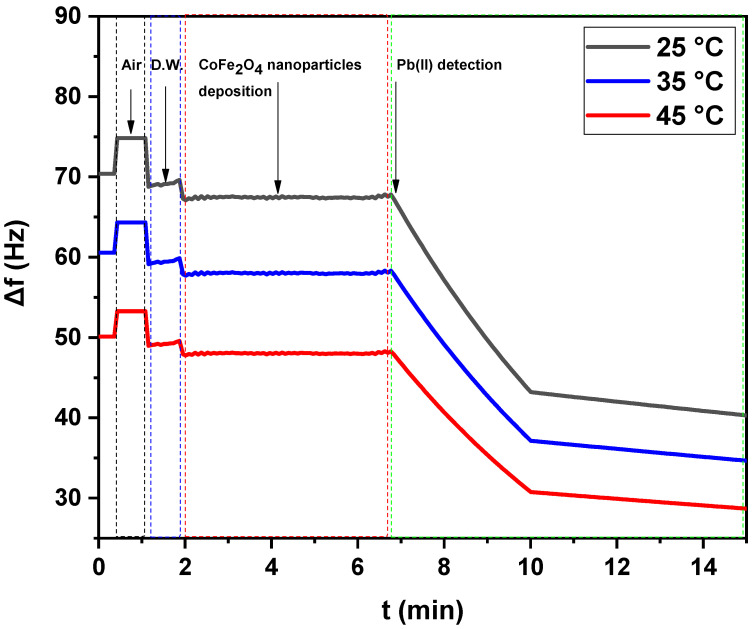
The net frequency shifts of the green synthesized CoFe_2_O_4_ nanoparticles-coated QCM nanosensor in Pb(II) aqueous solutions with different temperatures (25 °C, 35 °C, and 45 °C) as a function of time.

**Figure 7:**
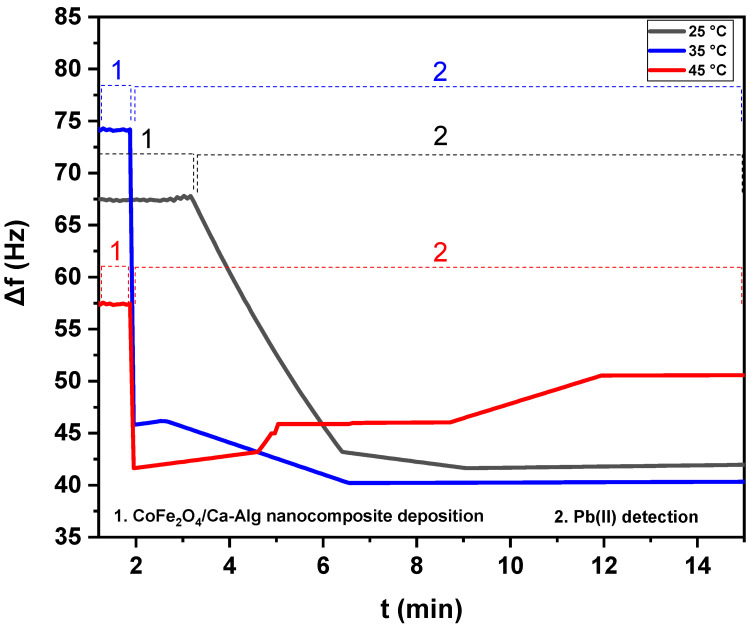
The net frequency shifts of the CoFe_2_O_4_/Ca-Alg nanocomposite-coated QCM nanosensors in Pb(II) aqueous solutions with different temperatures (25 °C, 35 °C, and 45 °C) as a function of time.

Figure 6 and Figure 7 show the net frequency shifts (Δ*f*) of the resonance frequency at the third overtone of the green synthesized CoFe_2_O_4_ nanoparticles and CoFe_2_O_4_/Ca-Alg nanocomposite-coated QCM nanosensors in Pb(II) aqueous solutions with various temperatures, i.e., 25 °C, 35 °C, and 45 °C vs. time, respectively. Note that the resonance frequency of the green synthesized CoFe_2_O_4_ nanoparticles and CoFe_2_O_4_/Ca-Alg nanocomposite in double-distilled water were taken as the reference state for calculating the frequency shift in response to the Pb(II) ions. It can be seen that the resonance frequency decreased when exposing the green synthesized CoFe_2_O_4_ nanoparticles and CoFe_2_O_4_/Ca-Alg nanocomposite-coated QCM quartz crystal to the Pb(II) aqueous solution, indicating the adsorption or complexation of Pb(II) onto the nanosensors surfaces. It was found that the 125 ng Pb(II) in the aqueous solutions can lead to frequency shifts of 27.49 ± 0.81, 23.63 ± 0.90, and 19.57 ± 0.86 Hz (Δ*f*) for the quartz crystal coated with green synthesized CoFe_2_O_4_ nanoparticles thin films and 25.85 ± 0.85, 33.87 ± 0.73, and 6.87 ± 0.08 Hz (Δ*f*) for the quartz crystal coated with CoFe_2_O_4_/Ca-Alg nanocomposite thin films in a real-time of about 11, 13, and 13 min at 25 °C, 35 °C, and 45 °C, respectively. Increasing the Pb(II) solution temperature resulted in more adsorption of Pb(II) ions, leading to larger frequency shifts. Thus, the nanosensors had a higher frequency response in Pb(II) aqueous solution with higher temperatures due to increasing the loaded masses of adsorbed Pb(II) ions. Accordingly, the sensing efficiency of Pb(II) increased gradually from 25 °C to 45 °C. From the obtained results, temperature increase enhances the mobility of Pb(II) ions and decreases the retarding force that acts on the diffusing ions. This results in the enhancement of the sorptive capacity of the adsorbent, an increase in chemical interaction between adsorbate and adsorbent, and the generation of active surface centers on an enhanced rate of intraparticle diffusion of Pb(II) ions into the pores of adsorbent at the higher temperatures.

For the temperature of 45 °C in the case of CoFe_2_O_4_/Ca-Alg nanocomposite, when the equilibrium in adsorption and complexation was clearly reached, partial desorption was observed. It was understandable because parts of Pb(II) ions were absorbed via the chemical complexation between CoFe_2_O_4_/Ca-Alg nanocomposite units and Pb(II) ions, where parts of Pb(II) ions may be only physically adsorbed. The physically adsorbed Pb(II) will be easily released away via the running water during a temperature increase. On the other hand, the process of chemical complexation was also in a dynamic equilibrium state. The continuous flushing of Pb(II) solution at high temperatures may also flush away parts of the Pb(II) complex that formed with CoFe_2_O_4_/Ca-Alg nanocomposite units. Additionally, this phenomenon may occur due to the overlapping of adsorption sites as a result of overcrowding of adsorbent particles and decreased adsorption, which was the major cause of the reduction in the contact surface of CoFe_2_O_4_/Ca-Alg nanocomposite for the removal of metal ions. By considering these results, the frequency shift of the adsorption step was selected to present the frequency response of green synthesized CoFe_2_O_4_ nanoparticles and CoFe_2_O_4_/Ca-Alg nanocomposite-coated QCM nanosensors in the aqueous solutions of Pb(II) ions. The schematic curves of Pb(II) detection by CoFe_2_O_4_ nanoparticles and CoFe_2_O_4_/Ca-Alg nanocomposite-QCM nanosensors included in the Supplementary Materials provide more information (Figures S1–S3).

**Figure 8:**
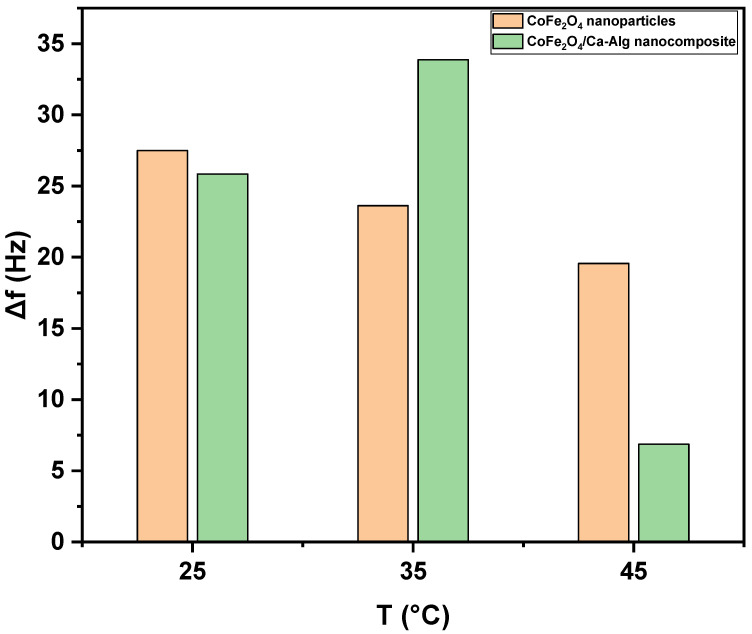
The net frequency shifts of the green synthesized CoFe_2_O_4_ nanoparticles and CoFe_2_O_4_/Ca-Alg nanocomposite-coated QCM nanosensors in Pb(II) aqueous solutions as a function of the solution temperature.

Figure 8 shows the frequency shifts response of green synthesized CoFe_2_O_4_ nanoparticles and CoFe_2_O_4_/Ca-Alg nanocomposite-coated QCM nanosensors in individual detected Pb(II) aqueous solutions with different temperatures. A decrease in frequency shift was observed with increasing the solution temperature. The lowest detection limit of green synthesized CoFe_2_O_4_ nanoparticles and CoFe_2_O_4_/Ca-Alg nanocomposite-coated QCM nanosensor for Pb(II) ions can reach as low as 125 ng/L in the aqueous solutions. The 125 ng/L Pb(II) ions in the aqueous solution at 25 °C led to the frequency shift of 27.49 ± 0.81 Hz (Δ*f*) for green synthesized CoFe_2_O_4_ nanoparticles thin film and 25.85 ± 0.85 Hz (Δ*f*) for CoFe_2_O_4_/Ca-Alg nanocomposite, respectively. It was decreased to 19.57 ± 0.86 Hz (Δ*f*) for green synthesized CoFe_2_O_4_ nanoparticles and 6.87 ± 0.08 Hz (Δ*f*) for CoFe_2_O_4_/Ca-Alg nanocomposite at 45 °C, respectively. Moreover, the resonance frequency change curve reveals the good adsorbed of Pb(II) ions on the surface of CoFe_2_O_4_/Ca-Alg nanocomposite than CoFe_2_O_4_ nanoparticles. The most superior sense ability of CoFe_2_O_4_/Ca-Alg nanocomposite towards low limit concentration of Pb(II) ions may be attributed to its higher surface area, more available active sits, porosity/diffusion nature, swelling capacity, and presence of carboxylic groups in the side groups as electron donors, which can easily form complexes with Pb(II) ions, and finally, its negative charge density that higher than of CoFe_2_O_4_ nanoparticles as estimated by the ζ-potential experiment, which enables it to attract Pb(II) ions by electrostatic interaction.

## 4. Conclusions

In this study, a novel green synthesis of CoFe_2_O_4_ nanoparticles using clove (*Syzygium aromaticum*) leaf extract was achieved. Then the synthesized nanoparticles were functionalized with Ca-alginate biopolymer to yield the CoFe_2_O_4_/Ca-Alg nanocomposite. The synthesized nanoparticles and nanocomposite underwent characterization of crystallinity, size, and shape using several tools such as XRD, N_2_ adsorption–desorption, DLS, ζ-potential, AFM, SEM, TEM, and EDX. Subsequently, the prepared nanoparticles and nanocomposite were used to fabricate nanosensors based on the QCM technique for reliable and rapid detection of low concentrations of Pb(II) ions in the aqueous solutions at different temperatures. The results exhibited frequency shift responses as follows; 27.49 ± 0.81, 23.63 ± 0.90, and 19.57 ± 0.86 Hz (Δ*f*) and 25.85 ± 0.85, 33.87 ± 0.73, and 6.87 ± 0.08 Hz (Δ*f*) for the quartz crystal coated with green synthesized CoFe_2_O_4_ nanoparticles thin films and CoFe_2_O_4_/Ca-Alg nanocomposite thin films in a real-time of 11, 13, and 13 min at 25 °C, 35 °C, and 45 °C, respectively, for a solution with a concentration of 125 ng Pb(II) ions. Furthermore, the CoFe_2_O_4_/Ca-Alg nanocomposite-coated QCM nanosensor displayed more advanced sensing for Pb(II) ions than the CoFe_2_O_4_ nanoparticles-coated QCM nanosensor.

## Figures and Tables

**Table 1:** Represents the BET surface area and porosity properties of green synthesized CoFe_2_O_4_ nanoparticles and CoFe_2_O_4_/Ca-Alg nanocomposite.

Material	Surface Area (S_BET_), m^2^/g	Total Pore Volume, cc/g	Average Pore Size, nm	Micropore Volume, cc/g (DA Method)	Average Micropore Size, nm (DA Method)
CoFe_2_O_4_ nanoparticles	63.21	0.068	2.17	0.066	1.55
CoFe_2_O_4_/Ca-Alg nanocomposite	34.05	0.083	4.86	0.047	1.17

## Data Availability

All the results and data used to support the findings of this study are included in the article.

## Supplementary Materials

The following supporting information can be downloaded at: https://www.mdpi.com/article/10.3390/nano12203620/s1, Figure S1: Illustrates resonance frequency changes in the curve of air, water, and CoFe_2_O_4_ nanoparticles in which resonance frequency decrease divided into three parts; the first one is the stable baseline frequency for empty QCM due to flow of air, the second step is due to the flow of water, and the third step is due to the deposition of CoFe_2_O_4_ nanoparticles on QCM chip with different temperature; Figure S2: Illustrates the resonance frequency changes curve which is divided into two parts; the left one is the stable baseline resonance frequency for CoFe_2_O_4_ nanoparticles precipitation on the QCM chip, and the gently slop part illustrates the beginning of Pb(II) adsorbing on the surface of CoFe_2_O_4_ nanoparticles and right part represents the complete deposition of Pb(II) ions on the CoFe_2_O_4_ nanoparticles surface at different temperature; Figure S3: Illustrates the resonance frequency changes in the curve of CoFe_2_O_4_/Ca-Alg nanocomposite at (A) 45 °C, (B) 25 °C, and (C) 35 °C as a result of deposition of the CoFe_2_O_4_/Ca-Alg nanocomposite and adsorption of Pb(II) ions on the QCM detector.
