# Supplementary material for: Design of a Novel Nanosensors Based on Green Synthesized CoFe2O4/Ca-Alginate Nanocomposite-Coated QCM for Rapid Detection of Pb(II) Ions
Source: Nanomaterials (Basel). 2022 Oct 15;12(20):3620. doi: 10.3390/nano12203620 (PMC9610289; doi:10.3390/nano12203620)
Supplement: Supplementary file 1 [file nanomaterials-12-03620-s001.zip › nanomaterials-1949896-supplementary.pdf]

nanomaterials

MDPI

Supplementary data

Design of a Novel Nanosensors Based on Green Synthesized $CoFe_2O_4$/Ca-Alginate Nanocomposite-Coated QCM for Rapid Detection of Pb(II) Ions

Wafa Al-Gethami [1], Dalal Alhashmialameer [1], Noha Al-Qasmi [1], Sameh H. Ismail [2,*] and Ahmed H. Sadek [2,3,*]

[1] Chemistry Department, Faculty of Science, Taif University, Al-Hawiah, Taif City P.O. Box 11099, Saudi Arabia
[2] Faculty of Nanotechnology for Postgraduate Studies, Sheikh Zayed Campus, Cairo University, 6th October City, Giza 12588, Egypt
[3] Zewail City of Science, Technology and Innovation, 6th October City, Giza 12578, Egypt
* Correspondence: drsameheltayer@yahoo.com (S.H.I.); ahsadek@zewailcity.edu.eg (A.H.S.)

The resonance frequency curve illustrates the good precipitation of $CoFe_2O_4$ nanoparticles and $CoFe_2O_4$/Ca-Alg nanocomposite on the surface of the QCM chip (decreasing in resonance frequency value followed by stable baseline (Figure (S1)). Then a stream of lead ions dissolved in double deionized water flows above the $CoFe_2O_4$ nanoparticles and $CoFe_2O_4$/Ca-Alg nanocomposite (Suddenly decreasing in resonance frequency value, which increased but still less the first one. Finally, after 15 minutes, a stable frequency baseline was reached, which indicates the maximum capacity sensing of lead ions. The curve shows the flow of air followed by doubled deionized water and finally followed by the deposition of $CoFe_2O_4$ nanoparticles or $CoFe_2O_4$/Ca-Alg nanocomposite until a baseline of the resonance frequency becomes stable. After the formation of thin film from $CoFe_2O_4$ nanoparticles or $CoFe_2O_4$/Ca-Alg nanocomposite, a solution of lead ions flows above the thin film surface at temperatures of 25, 35, and 45 °C. In Figures S1 and S2, the resonance frequency changes the curve of the $CoFe_2O_4$ nanoparticles sensor indicating no important changes by changing temperature. However, a decrease in resonance frequency value indicates the adsorption of lead ions on the surface of $CoFe_2O_4$ nanoparticles as shown in Figure (S1). The resonance frequency changes curve of $CoFe_2O_4$/Ca-Alg nanocomposite illustrates the achieving of different sense capacities with the change in the temperature value. However, the resonance frequency changes curve at 25 °C illustrates the normal adsorption of lead ions on the surface of $CoFe_2O_4$/Ca-Alg nanocomposite (gently linear decreasing in the resonance frequency curve). The resonance frequency changes at 35 °C illustrate the dramatic change in resonance frequency values, whereas the suddenly extensive decrease indicates high-speed adsorption of lead ions on the nanocomposite surface with a stable baseline for 3.5 minutes followed by an increase in the resonance frequency of the normal adsorbed lead ions on the surface of $CoFe_2O_4$/Ca-Alg nanocomposite, which indicates leaching out of some lead ions for 4 minutes. Then, the previous stages are followed by another increase in the resonance frequency value, which indicate the second leaching out of lead ions from the surface of the nanocomposite. Finally, the stable resonance frequency baseline was continued to the end of the experiment (about 6 minutes), which indicates the maximum capacity sensing of lead ions by $CoFe_2O_4$/Ca-Alg nanocomposite. The zone in Figure (S3) represents the gently linear decrease in resonance frequency changes. The resonance frequency changes at 45 °C illustrate the dramatic change in resonance frequency value, where the suddenly extensive decrease indicates high-speed adsorption of lead ions on the nanocomposite surface with a stable baseline reached for 1.5 minutes and followed by another decrease in the resonance frequency, which indicates more adsorption of lead ion on the surface of the nanocomposite at 45 °C rather than at both 25 and 35 °C. However, the decrease in resonance frequency continued

for about 4 minutes. Finally, the resonance frequency value remains stable until the end of the experiment as shown in Figure (S3).

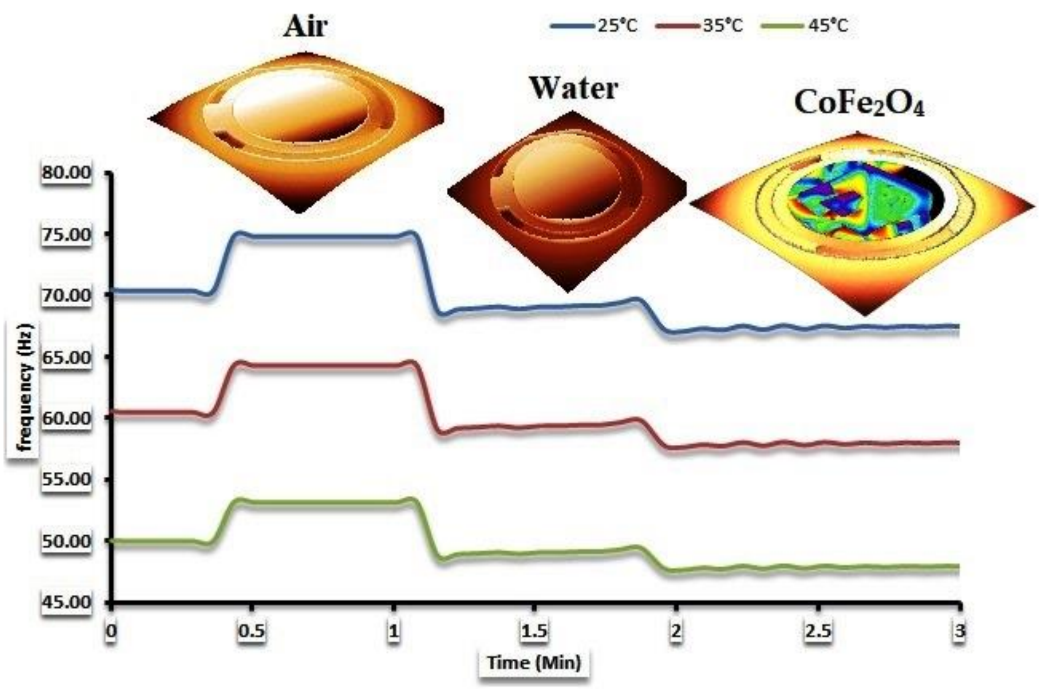

Figure S1. Illustrates resonance frequency changes in the curve of air, water, and $CoFe_2O_4$ nanoparticles in which resonance frequency decrease divided into three parts; the first one is the stable baseline frequency for empty QCM due to flow of air, the second step is due to the flow of water, and the third step is due to the deposition of $CoFe_2O_4$ nanoparticles on QCM chip with different temperature.

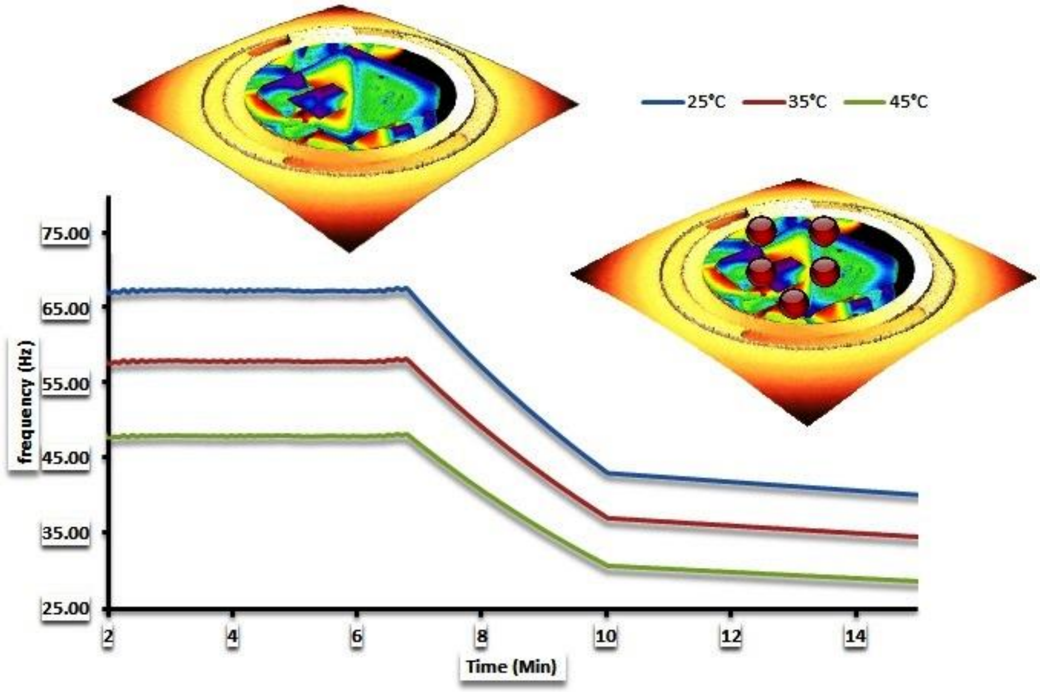

Figure S2. Illustrates the resonance frequency changes curve which is divided into two parts; the left one is the stable baseline resonance frequency for $CoFe_2O_4$ nanoparticles precipitation on the QCM chip, and the gently slop part illustrates the beginning of Pb(II) adsorbing on the surface of $CoFe_2O_4$ nanoparticles and right part represents the complete deposition of Pb(II) ions on the $CoFe_2O_4$ nanoparticles surface at different temperature.

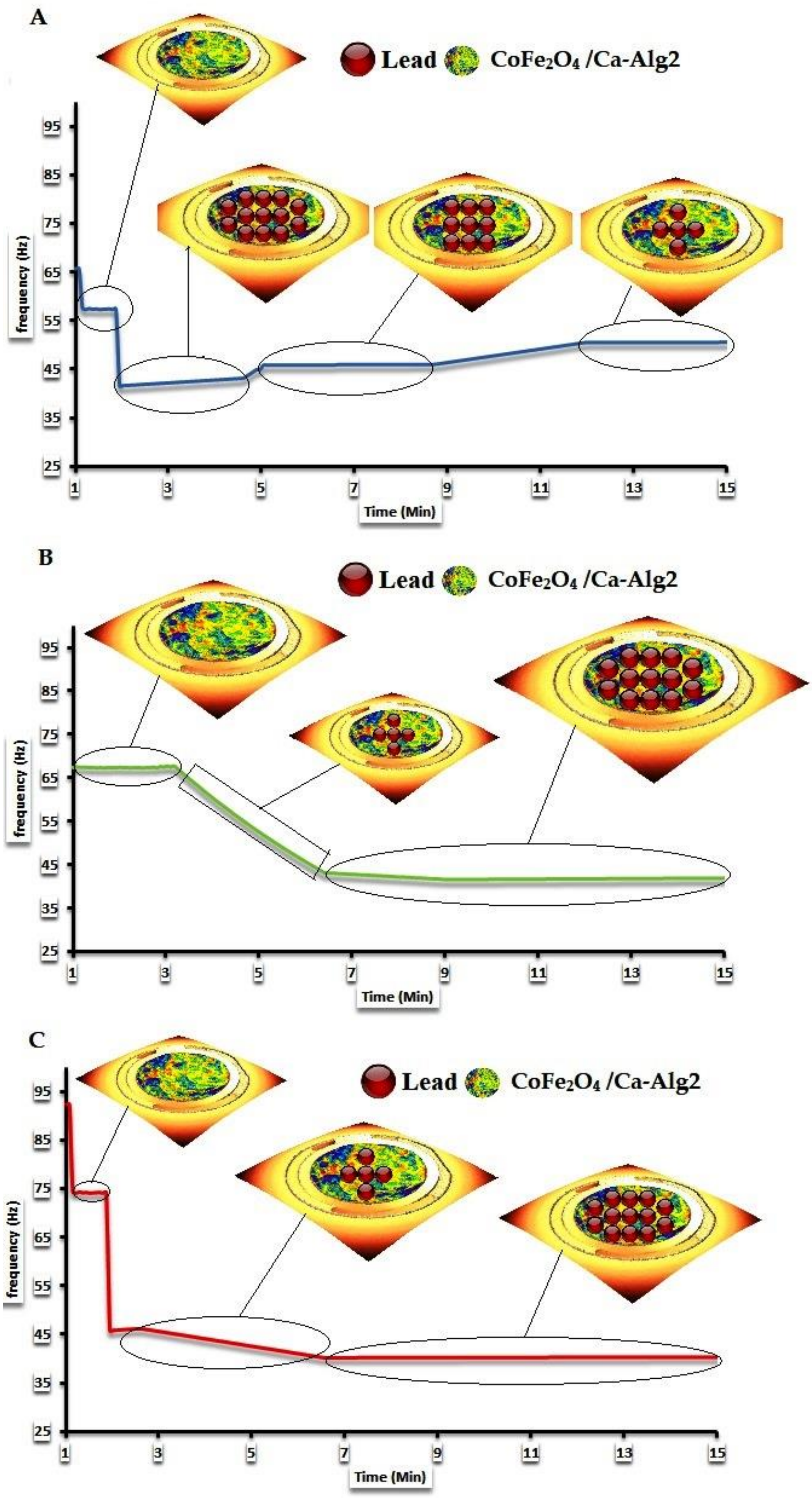
A
Lead
CoFe2O4 /Ca-Alg2
frequency (Hz)
95
85
75
65
55
45
35
25
1
3
5
7
9
11
13
15
Time (Min)
B
Lead
CoFe2O4 /Ca-Alg2
frequency (Hz)
Time (Min)
C
Lead
CoFe2O4 /Ca-Alg2
frequency (Hz)
Time (Min)

Figure S3. Illustrates the resonance frequency changes in the curve of $CoFe_2O_4$/Ca-Alg nanocomposite at (A) 45 °C, (B) 25 °C, and (C) 35 °C as a result of deposition of the $CoFe_2O_4$/Ca-Alg nanocomposite and adsorption of Pb(II) ions on the QCM detector.
